# Hypoxia and re-oxygenation effects on human cardiomyocytes cultured on polycaprolactone and polyurethane nanofibrous mats

**DOI:** 10.1186/s13036-024-00432-5

**Published:** 2024-06-06

**Authors:** Zuzanna Iwoń, Ewelina Krogulec, Aleksandra Kierlańczyk, Michał Wojasiński, Elżbieta Jastrzębska

**Affiliations:** 1 Chair of Medical Biotechnology, Faculty of Chemistry, Warsaw University of Technology, Warsaw, Poland; 2 Laboratory of Cell Signaling and Metabolic Disorders, Nencki Institute of Experimental Biology PAS, Warsaw, Poland; 3 Department of Biotechnology and Bioprocess Engineering, Faculty of Chemical and Process Engineering, Warsaw University of Technology, Warsaw, Poland; 4 Centre for Advanced Materials and Technologies, CEZAMAT Warsaw University of Technology, Warsaw, Poland

**Keywords:** Nanofibrous scaffolds, Solution blow spinning, Human cardiac cells, Hypoxia, Hypoxia with re-oxygenation, Tissue engineering

## Abstract

Heart diseases are caused mainly by chronic oxygen insufficiency (hypoxia), leading to damage and apoptosis of cardiomyocytes. Research into the regeneration of a damaged human heart is limited due to the lack of cellular models that mimic damaged cardiac tissue. Based on the literature, nanofibrous mats affect the cardiomyocyte morphology and stimulate the growth and differentiation of cells cultured on them; therefore, nanofibrous materials can support the production of in vitro models that faithfully mimic the 3D structure of human cardiac tissue. Nanofibrous mats were used as scaffolds for adult primary human cardiomyocytes (HCM) and immature human induced pluripotent stem cell-derived cardiomyocytes (iPSC-CMs). This work focuses on understanding the effects of hypoxia and re-oxygenation on human cardiac cells cultured on polymer nanofibrous mats made of poly(ε-caprolactone) (PCL) and polyurethane (PU). The expression of selected genes and proteins in cardiomyocytes during hypoxia and re-oxygenation were evaluated. In addition, the type of cell death was analyzed. To the best of our knowledge, there are no studies on the effects of hypoxia on cardiomyocyte cells cultured on nanofibrous mats. The present study aimed to use nanofiber mats as scaffolds that structurally could mimic cardiac extracellular matrix. Understanding the impact of 3D structural properties in vitro cardiac models on different human cardiomyocytes is crucial for advancing cardiac tissue engineering and regenerative medicine. Observing how 3D scaffolds affect cardiomyocyte function under hypoxic conditions is necessary to understand the functioning of the entire human heart.

**Supplementary Information:**

The online version contains supplementary material available at 10.1186/s13036-024-00432-5.

## Introduction

Most heart diseases such as heart failure, arrhythmia, and myocardial infarction (MI) are induced by a chronically insufficient amount of oxygen (hypoxia) supply of cardiomyocytes (CMs), whereby cells undergo damage and apoptosis [1]. The main cause of damage to the heart’s cells is limited blood supply with oxygen and nutrients (ischemia) caused by blockage of the vessels through deposition of lipids, such as cholesterol, in the wall of the coronary arteries. Additionally, cardiomyocytes do not have the ability to regenerate. Damaged CMs are replaced by cardiac fibroblasts, where scar tissue is formed. Scar tissue formation reduces the heart’s ability to contract correctly, leading to abnormal heart functioning [2, 3].

Currently, available treatments for damaged cardiac tissue include drugs (anticoagulants, beta-blockers, calcium channel blockers, anti-thrombotic therapy) or special devices that support cardiac functioning (pacemakers, artificial heart, coronary artery bypass) [4]. Unfortunately, in many cases, using these solutions is inadequate, as they only provide a temporary improvement in the condition of the patients. Nowadays, the best treatment for heart disease is transplantation. However, the number of potential donors is insufficient due to the increasing number of patients suffering from cardiac diseases. For this reason, it is necessary to search for new therapies that will promote cardiac regeneration and reduce the number of patients requiring transplantation [5].

Ischemia induces hypoxia, which can cause cardiomyocyte dysfunction, disorders of metabolic pathways, and apoptotic death of cardiac cells after a short time. However, research is focused on hypoxia-related processes because they are not entirely known [6]. In addition, a growing number of studies indicate that the main effect on hypoxia-induced damage to cardiac cells is the delivery of oxygen back to the CMs, called re-oxygenation in in vitro models or when blood is redelivered to the cells (reperfusion) in vivo [7–10]. For example, Xu et al. carried out hypoxia (H) and re-oxygenation (R) (H/R by 6 h/12 h) on rat cardiomyoblasts (H9c2) to confirm the influence of myosin 1b (myo1b) on cell apoptosis and autophagy. Thus, the strategy limiting the promotion of myo1b may be the prevention of cardiac damage [9]. In other research, scientists compared the cardioprotective abilities of miRNA for immortalizing mouse cardiomyocytes HL-1 after H/R (2 h/24 h) and for mouse myocardial after 30 min ischemia and 1 h reperfusion (I/R) and for both models, the cardioprotective abilities of miRNA were confirmed [10]. Therefore, studying the effects of hypoxia and hypoxia/re-oxygenation cardiac cells on in vitro and in vivo models is crucial for understanding and comparing the various models of damaged cardiomyocytes [11]. It supports the search for an effective method of treating damaged heart cells. Most in vitro studies are conducted on 2D models, and in vivo studies are conducted on rodent model organisms, which are not adequate models. To our knowledge, there are no accurate models of the complexity of human heart tissue after ischemia/reperfusion [12–15].

Research into the regeneration of a damaged human heart is limited due to the lack of cellular models that mimic damaged cardiac tissue; therefore, it is necessary to develop a human cardiac tissue model that will enable the mimic of disease states in vitro. Hence, new methods are being looked for, and one technique that can potentially mimic appropriate human cellular models of the heart is tissue engineering (TE) [16]. TE combines knowledge of biomaterials, engineering, biology, physics, and medicine [17]. Tissue engineering involves the development of tissue models that can be used for diagnostic purposes to test drug efficacy and toxicity in vitro [16, 17]. Scaffolds such as nanofibers are used to graft and culture cells. Various nanofibrous mats with the required appropriate physicochemical properties, such as sufficient elasticity to allow the cells to contract, fiber diameter to mimic the fibers that build the extracellular matrix (ECM), a high surface-to-volume ratio, biocompatibility, and the ability to obtain fibers with a parallel arrangement as it is in cardiac tissue, are utilized. The nanofibers can also be electrically conductive, allowing researchers to simulate electrophysiological conditions in cardiac tissue [18]. Several methods of manufacturing nanofibers are known, among which the most important are electrospinning (ES) and solution blow spinning (SBS). The techniques allow the use of natural materials (e.g., collagen, gelatin) and synthetic materials such as polyurethane (PU) and poly(ε-caprolactone) (PCL) as well as composites [19–21]. Nanofibers have been used so far as a scaffold for human cardiac cell attachment, viability, and maturation [16–24]. For example, Zhang et al. produced electrospun PCL nanofibrous mats with modified surfaces by gelatin solution for the maturation of human induced pluripotent stem cell-derived cardiomyocytes (iPSC-CMs). Scientists confirmed the high viability of cells growing on nanofibrous mats, increased expression of genes and proteins characteristic of mature cardiomyocytes such as cTnT (cardiac troponin T), a-actinin, MYL2 (myosin light chain 2), increased β-MHC/α-MHC (myosin heavy chain β and α), and MLC2v/MLC2a (myosin light chain 2a and 2v) ratios [23]. In addition, it was noted that the cells grown on nanofibers showed enhancing calcium transient kinetics, confirming that iPSC-CMs grown on PCL nanofibers are closer to adult human cardiomyocytes than cells grown on PS plate [23].

It has been investigated that nanofibrous mats increase cell viability, rod-like cell morphology, and parallel orientation, which is similar to cardiomyocytes in the human cardiac tissue. Their usage may also support the differentiation of stem cells into cardiomyocytes. For this reason, nanofibers have great potential for use in tissue engineering as a model to study cardiac treatments and heart disease (mainly caused by hypoxia). To date, there is a lack of research on using nanofiber mats as a biomaterial for studying hypoxic human heart cells. Cardiomyocyte function under hypoxia or hypoxia with re-oxygenation has been compared for iPSC-CM cultured in 2D and 3D (using) hydrogel cultures [25, 26]. There is still a lack of information on whether there are differences in the cellular response to the hypoxia and re-oxygenation state depending on the human cardiac cell line used, or whether the cellular response is organism specific.

This study aimed to use nanofibrous mats (made of PCL and PU) as scaffolds that mimic in vivo conditions to investigate the effects of hypoxia and re-oxygenation on human cardiac cells cultured on 3D structures. We decided to use PCL and PU nanofibrous mats because, so far, studies proved their usage as 3D cardiac tissue models [20, 22, 27–30]. PCL and PU are non-toxic, biodegradable polymers with appropriate mechanical strength and elasticity for culture cardiac cells [31, 32]. In contrast to other works, we used nanofibrous mats with different physicochemical properties (types of polymer, elasticity) to study the influence of functioning human cardiomyocytes after hypoxia or hypoxia with re-oxygenation. Differences in the functioning of human cardiomyocytes cultured on nanofibrous mats under pathological conditions (hypoxia or H/R) were established. Cell response on hypoxia conditions could differ between 2D and 3D cell models. The study used adult primary human cardiomyocytes (HCM) and immature human induced pluripotent stem cell-derived cardiomyocytes. Although iPSC-CMs are immature, studying their response to hypoxia and hypoxia with re-oxygenation in 3D models may support further research into their use in the production of specialized 3D heart models, such as organoids. In addition, to the best of our knowledge, this is the first study in which nanofiber mats have been used to study the response of human cardiac cells to pathological conditions. The cellular response to hypoxia and re-oxygenation conditions was studied in human and chimpanzee iPSC-CM cells [33]. The responses to hypoxic and re-oxygenation conditions were similar between species. In this study, we asked whether we would observe similar responses to hypoxia and H/R between human cardiomyocytes derived from induced stem cells and the primary human HCM cell line. Since both lines are derived from humans, one would potentially expect an analogous response at the cellular level. However, iPSC-CMs are immature cells, while the HCM line is cells derived from an adult organism. Since iPSC-CMs are increasingly used as model cells to study cardiac function, knowing how they differ from adult-derived mature cardiac cells is crucial. To the best of our knowledge, there are no comparison studies on the effects of hypoxia on human mature and immature cardiomyocytes cultured on nanofibrous mats. We can assume that due to the special properties of nanofibers, using them as a structural element for cells after hypoxia will allow the cellular response to be more similar to their in vivo response.

## Materials and methods

### PCL and PU nanofibrous mats

Poly(ε-caprolactone) (PCL, Sigma Aldrich Mn = 80 000) nanofibers with an average diameter of 509 ± 178 nm, and polyurethane (PU, ChronoFlex C75D, AdvanSource Biomaterials) nanofibers with an average diameter of 452 ± 151 nm were produced with the solution blow spinning method (SBS). The production method and physicochemical characterization of nanofibrous materials were described in our previous study [34]. Additionally, the characterization of the nanofibrous mats is given in Supplementary Materials in Figure S2.

### Cell culture

Human cardiomyocytes HCM (ScienCell) were cultured in polystyrene (PS) flasks covered by 0.01% poly-l-lysine solution. HCM cells were cultured in Dulbecco’s Modified Eagle Medium: Nutrient Mixture F-12 (DMEM/F12, Gibco) supplemented with 10% v/v fetal bovine serum (FBS, Gibco), 1% v/v 100 mM Penicillin–Streptomycin (Sigma-Aldrich), 1% v/v 200 mM L-glutamine (Sigma-Aldrich), 1% v/v 100 mM sodium pyruvate (Sigma-Aldrich), 1% v/v cardiac myocyte growth supplement (CMGS, ScienCell) and 0.01% v/v MEM non-essential amino acids (NEAA, Sigma-Aldrich). When the cells have received a confluence of more than 90%, wash them with phosphate-buffered saline (PBS, Sigma-Aldrich) and trypsinized with 0.25% Trypsin (Sigma-Aldrich), then passaged or seeded on nanofibers. The cultures were maintained in a humidified incubator (37 °C, 5% CO_2_). The cells for the sixth passage were used in the experiments (based on the supplementary materials, Fig. S1, it was estimated the cells for experiments up to 6–8 passages can be used).

Human induced pluripotent stem cells (iPSCs, IIMCBi001-A (ELE10) line) were received from the Laboratory of Molecular and Cellular Neurobiology of the International Institute of Molecular and Cell Biology in Warsaw. The protocol for obtaining iPSCs was described by Liszewska et al. [35]. Differentiation of hiPSCs into iPSC-CMs was conducted based on the GiWi protocol described by Lian et al. [36]. The process is based on the modulation of the pathway with the GSK3 inhibitor and Wnt inhibitor. iPSC-CMs were washed with Dulbecco’s phosphate-buffered saline (DPBS, ATCC) and detachment by TrypLE (Thermo Fisher Scientific). The cells were resuspended in RPMI medium with 20% v/v fetal bovine serum (FBS, Gibco), 1% v/v 100 mM Penicillin–Streptomycin (Sigma-Aldrich*)* and 5 µM ROCK inhibitor. iPSC-CMs were seeded on the prepared nanofibrous mats and polystyrene plate (coated with 0.1% gelatin solution). After 48 h, the medium is replaced by RPMI with added B-27 with insulin (Thermo Fisher Science) by 24 h. The cultures were maintained in a humidified incubator (37 °C, 5% CO_2_).

### Human cardiomyocytes on nanofibrous mats

Nanofibrous mats were placed in 24-well plates, sterilized with 70% EtOH (POCH) for 30 min, and then dried in an oven at 40 °C (Binder). Next, the surface of nanofibrous mats was modified with oxygen plasma (0.3 mbar, 90 s, Diener) and coated with protein solutions to improve their hydrophilic properties. Nanofibrous mats were coated with 0.01% poly-l-lysine solution (ScienCell) and 0.1% gelatin solution (Sigma-Aldrich) for the culture of HCM and iPSC-CMs, respectively. 24 h after that, HCM and iPSC-CM cells were seeded with densities of 6.6 × 10^4^ cells/cm^2^ and 2 × 10^5^ cells/cm^2^, respectively. Images of HCM and iPSC-CMs grown on PCL and PU nanofibrous mats are added in the Supplementary Materials (Figure S3 and Figure S4).

### Hypoxia induction and re-oxygenation

A study of the effect of hypoxia on HCM and iPSC-CM cells was carried out by placing the cells in an incubator (Thermo Fisher Scientific) designed for hypoxia equipped with an oxygen level sensor. For primary human cardiomyocytes, the medium was changed to serum-free, glucose-free, and phenol-free Dulbecco’s Modified Eagle Medium (DMEM, Gibco). For human pluripotent stem cell-derived cardiomyocytes, the medium was modified on serum- and glucose-free DMEM (Gibco) containing 1% MEM NEAA (Aldrich-Sigma), 1% GlutaMAX (Gibco) and 1% penicillin/streptomycin (Sigma-Aldrich). Hypoxic media for both types of cells were pre-incubated in an environment without oxygen for 24 h to remove the dissolved oxygen. Then, cells were maintained under hypoxic conditions at 1% O_2_, 5% CO_2_, and 37 °C for 6 h. The cells cultured under normoxia conditions (21% O_2_, 5% CO_2_, and 37 °C) on polystyrene plates were used as a control. Hypoxia with re-oxygenation (H/R) was obtained by replacing the hypoxia with standard media in cell culture. The cells undergoing hypoxia and re-oxygenation were placed in an incubator (21% O_2_, 5% CO_2_, and 37 °C) for 24 h.

### Immunostaining fluorescence

Cells after hypoxia and hypoxia with re-oxygenation (H/R) were fixed with 4% paraformaldehyde for 10 min at room temperature (RT) and permeabilized with 0.5% Triton X-100 (Sigma-Aldrich) in DPBS. Next, samples were blocked with 2.4% bovine serum albumin (BSA, Thermo Fisher Scientific) solution in DPBS (50 min, RT). They were incubated with mouse anti-human monoclonal hypoxia-inducible factor 1-alpha (HIF-1α, Novus Bio) antibody (1:100) overnight at 4 °C. Then, the cells were stained with goat anti-mouse Alexa Fluor 488 (1:200, Thermo Fisher Scientific) and Phalloidin conjugated with Alexa Fluor-568 (1:400, Thermo Fisher Scientific) for 1 h at RT. Next, Hoechst 33,342 (10 µg/ml in DPBS) (Thermo Fisher Scientific) was added. After 5 min, the cells were washed with 0.1% Triton X-100. The stained cells were observed, and relative fluorescence intensity was determined under the Zeiss Axio Observer 7 + LSM 900 confocal microscope. Additionally, sarcomeric distribution was evaluated using the ImageJ software (version 1.54f).

### Apoptosis and necrosis assay

To evaluate the cell death induced by hypoxia and H/R, an Apoptosis/ Necrosis Assay Kit (Abcam) was used. The cells were labeled based on the manufacturer’s protocol by three fluorescence dyes: CytoCalcein Violet 450 (Ex/Em = 405/450 nm), Appoxin Green (Ex/Em = 490/525 nm), and 7-AAD (Ex/Em = 546/647 nm). CytoCalcein is a cell-permeable dye hydrolyzed into a strong fluorescence dye in living cells. Appoxin Green binds to phosphatidyl serine (PS) on the surface of the apoptotic cells. 7-AAD is an impermeable dye through the cell membrane, binds to the double strand of DNA, and labels cells in later apoptosis or necrosis stages. HCM and iPSC-CMs were observed under a microscope (Nikon Eclipse Ts2-FL). In addition, quantitative analysis of the percentage of viable, apoptotic, and necrotic cells was performed using ImageJ software.

### Real-time reverse transcription-quantitative polymerase chain reaction

Total RNA was extracted using RNeasy Mini Kit (Qiagen) and then reverse transcribed into cDNA using RevertAid H Minus First Strand cDNA Synthesis Kit (Thermo Fisher Scientific). RT-PCR was carried out utilizing SsoAdvanced Universal SYBR Green Supermix (Bio-Rad) on a CFX Connect Real-Time PCR System (Bio-Rad). GAPDH as a housekeeping gene was used. The sequences of the primers used to analyze the expression of human genes are given in the Supplementary Information (Table S1).

### Statistical analysis

Statistical significance was determined as the mean ± standard deviation (SD) by Student’s t-tests or ANOVA for three independent experiments using the OriginPro 8 software. Values of *p* < 0.05 were considered statistically significant and marked with an asterisk.

## Results

### Analysis of HIF-1α level and sarcomeric distribution after hypoxia and hypoxia/re-oxygenation

The study aimed to investigate the effect of hypoxia and hypoxia/re-oxygenation (H/R) on the function of cardiomyocytes cultured on 3D structures. The response of two types of human cardiac cells, a commercially available line of human cardiomyocytes - HCM and a line of human cardiomyocytes obtained by differentiating induced pluripotent stem cells - iPSC-CMs, was studied. The function of iPSC-CMs has not been fully investigated so far. However, thanks to their phenotype and spontaneous contraction, they can be a promising cell model in cardiovascular disease studies. Nanofibrous mats affect the cardiac cell morphology and stimulate growth and differentiation; therefore, they may also affect the response of cells to hypoxia. The study used polycaprolactone and polyurethane nanofibers with diameters of 509 ± 178 nm and 452 ± 151 nm, respectively. The nanofibers were oriented parallel to each other, and their Young’s modulus was 48.6 ± 3.6 MPa (for PCL) and 60.3 ± 8.9 MPa (for PU). Our previous research described the characterization of nanofiber’s morphology in detail [34, 37]. Nanofibrous mats were utilized as a substrate that can influence cardiac cell response under hypoxia, and the results were compared with the controls (cultures on polystyrene (PS)). We established three types of cultures on PS plates: the cells cultured under (a) normal oxygenation (21% O_2_) conditions as normoxia control, (b) under hypoxia for 6 h as hypoxia control, and under hypoxia for 6 h and re-oxygenation for 24 h as H/R control.

**Fig. 1:**
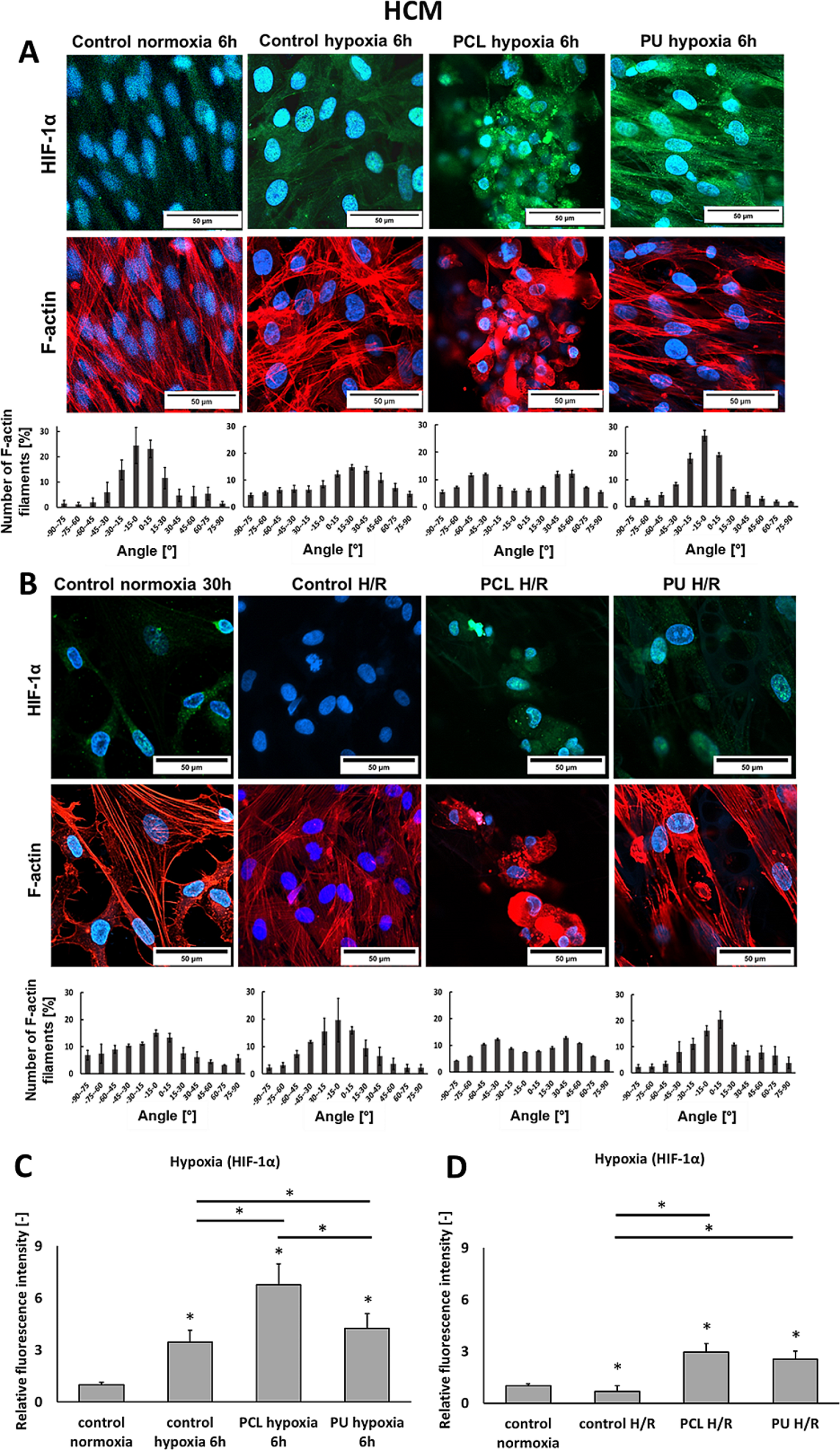
Immunofluorescence staining of F-actin (red), HIF-1α (green), and nucleus (blue) in HCM cells cultured on a polystyrene plate (control) and PCL and PU nanofibrous mats under normoxia (21% O_2_), 6 h hypoxia (1% O_2_) (**A**), and 6 h hypoxia + 24 h re-oxygenation. (**B**) The expression of HIF-1α of HCM cells (**C**) after hypoxia and (**D**) after hypoxia with re-oxygenation. *- *p* < 0.05- statistically significant differences compared to control normoxia, *with line-*p* < 0.05 statistically significant differences between groups. *n* > 3. Scale bar 50 μm

The results of human cardiomyocyte staining of F-actin and HIF-1α after hypoxia and re-oxygenation are shown in Fig. 1. A parallel arrangement cytoskeleton structure characterized HCM cells cultured on polystyrene plate (PS) under normoxia. The cells cultured on PS under hypoxia conditions for 6 h showed disrupted actin cytoskeleton structure and increased levels of HIF-1α factor (3.5-fold higher than in normoxia control). The cells cultured on PCL and PU nanofibrous mats subjected to 6 h of hypoxia also showed disrupted actin cytoskeleton structure and increased HIF-1α (for cultures grown on PCL scaffolds is 6.8 higher, for PU nanofibers is 4.3 than for normoxia). Despite disrupting the cytoskeleton structure, parallel orientation was evident in the cells cultured on PU nanofibrous mats. The cytoskeletal structure of the cells cultured on PCL nanofibers subjected to 6 h of hypoxia was more disturbed than observed for the other culture types. These results suggest that HCM cells, after hypoxia cultured on PCL, show greater sensitivity to hypoxic conditions than cultures grown on PU nanofibrous mats and PS.

Some literature reports indicate that the influence on damaged cardiac cells has delivered oxygen back to CMs after hypoxia [7–9]. Therefore, the influence of re-oxygenation on human cardiomyocyte function in 3D models was studied. After 6 h of hypoxia and 24 h of re-oxygenation (Fig. 1B), the cells had similar disruption of the cytoskeletal structure as the cells maintained 6 h under hypoxia. The most significant differences between each surface were observed for the HIF-1α. In control, H/R cells, HIF-1α gene expression decreased, while an increase was observed for HCMs cultured on PCL and PU nanofibrous mats. The protein level was 2.9-fold (for PCL) and 2.5-fold (for PU) higher than normoxia control. Hypoxia and re-oxygenation caused a decrease of HIF-1α for culture performed on PS plate (0.6-fold of 30 h normoxia control). This may indicate that nanofibrous mats can reduce the oxygen transfer rate into cells, affecting the high expression of HIF-1α. Slight disruption of F-actin filaments was observed in cells cultured on PU and PS nanofiber mats. In contrast, cells cultured on PCL nanofiber mats were characterized by significant disruption of the cytoskeletal structure, which may indicate a higher degree of cell damage. In Fig. 1C and D, the cells grown on nanofibrous mats under hypoxia and H/R show significantly higher HIFα expression than normoxia control. However, for H/R cultures, the expression is significantly lower than for cultures after hypoxia. Moreover, for the cells cultured on PCL nanofibrous mats, the disrupted structure of F-actin filaments persists for cultures after hypoxia and H/R. In contrast, the cellular structure returns to parallel arrangements for cultures grown on PS. HCMs grown on PU nanofibers remain aligned after hypoxia or hypoxia with re-oxygenation.

Morphological and physiological terms of iPSC-CMs are more similar to fetal than adult cardiomyocytes. Fetal cells rely on anaerobic metabolism, based on glycolysis, to a greater resistance to hypoxia than mature cells. For this reason, the cells adapt to anaerobic conditions and may be less sensitive to the negative effects of oxygen deprivation [38]. Based on the above, iPSC-CMs were used as a promising cell model in our studies.

**Fig. 2:**
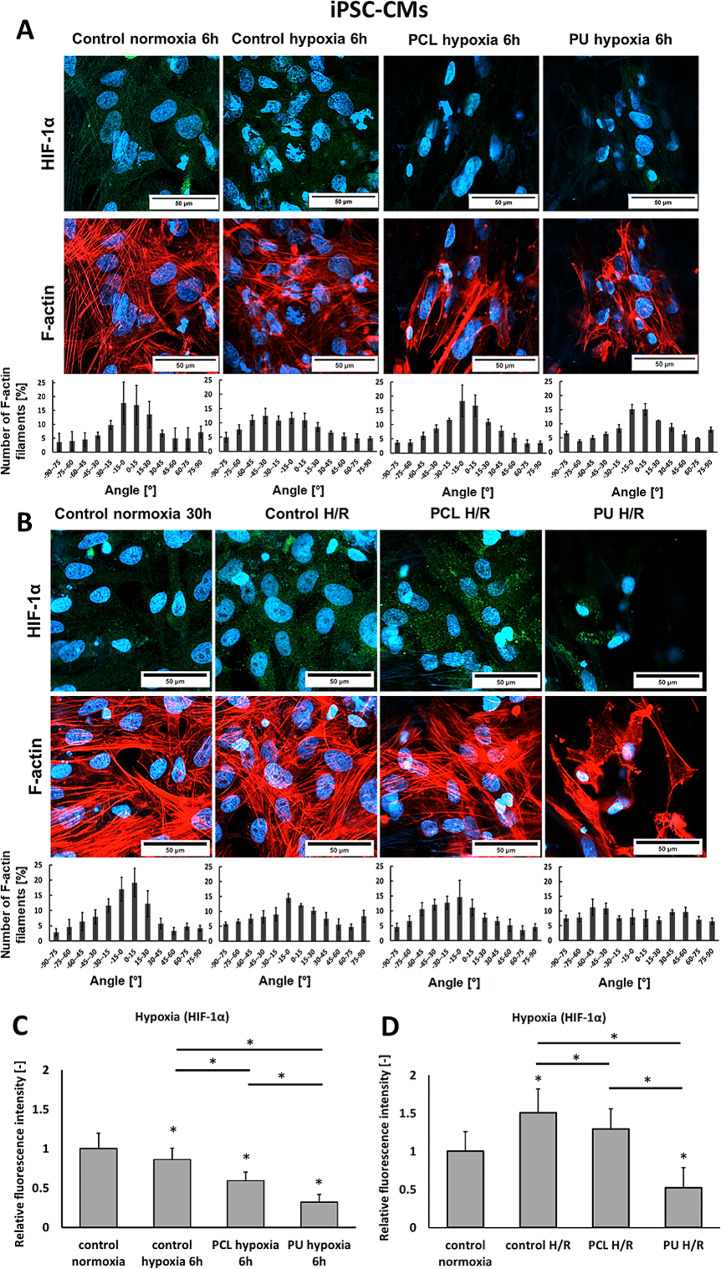
Immunofluorescence staining of F-actin (red), HIF-1α (green), and nucleus (blue) in iPSC-CM cells cultured on a polystyrene plate (control), and PCL and PU nanofibrous mats under normoxia (21% O_2_), 6 h hypoxia (1% O_2_) (**A**), and hypoxia 6 h + 24 h re-oxygenation. (**B**) The expression of HIF-1α of iPSC-CM cells (**C**) after hypoxia and (**D**) after hypoxia with re-oxygenation. *- *p* < 0.05- statistically significant differences compared to control normoxia, *with line-*p* < 0.05 statistically significant differences between groups. *n* > 3. Scale bar 50 μm

The results of iPSC-CM staining of F-actin and HIF-1α are shown in Fig. 2A and B. In the case of cells cultured on a polystyrene plate under normoxia, a parallel arrangement of F-actin filaments was observed, while in the case of cells subjected to hypoxia, disrupted sarcomeric fibers were noticed. This may indicate cell damage in response to hypoxia. A disrupted sarcomeric distribution also characterized the cells cultured on PCL and PU nanofibrous mats under hypoxia. The cytoskeletal structure of the cells cultured on nanofibrous mats made of PCL was characterized by a more parallel orientation than actin fibers building cells cultured on PU nanofibers. HIF-1α protein level decrease after 6 h of hypoxia was noted for both nanofibrous mats and PS after hypoxia compared to pluripotent stem cell-derived cardiomyocytes cultured in normoxia. It equaled 0.9-fold, 0.6-fold, and 0.3-fold of 6 h normoxia control for PS, PU, and PCL, respectively.

It was noticed that hypoxia and re-oxygenation also disrupted the actin microfilaments of iPSC-CM cells. The cells cultured on PS (control) and PCL nanofibrous mats after H/R showed more parallel arrangement than cells cultured on PU nanofibrous mats. Moreover, an increase of the HIF-1α was observed for the cells after H/R for PS and PCL nanofibrous mats compared to the cells maintained under normoxia (for PS was 1.5-fold and PCL was 1.3-fold of normoxia control). No significant differences have been noted when comparing the arrangement of actin filament in cultures after hypoxia and hypoxia with reoxygenation. In contrast, the level of HIF-1α protein changes significantly depending on the substrate and conditions (hypoxia or H/R).

In Fig. 2C and D, the HIF-1α level decreases significantly for all types of cultures after hypoxia, while for H/R, it increases significantly for cultures grown on PCL nanofibers and PS. For iPSC-CMs cultured on PU nanofibrous mats, there is also an increase in HIF-1α level after H/R, but it is significantly lower than the level of this protein in normoxia control. It seems likely that the observed increase in HIF-1a protein levels after 24-hour reoxygenation on nanofibers, relative to hypoxia without reoxygenation, may be related to the more difficult penetration of oxygen into the nanofiber structure and thus the delayed response of iPSC-CM cells to hypoxic conditions. To our knowledge, there is no explanation in the literature for this mechanism for immature cells cultured on 3D structures. However, this may be because of the impact of the physicochemical properties of the nanofibers on cell functioning.

Comparing the function of HCM and iPSC-CMs cultured on nanofibrous mats, it was noticed that actin damages after hypoxia and maintained in cultures after re-oxygenation. However, it has been noted that the disruption of actin filaments varies depending on the type of culture and nanofiber mats. HCMs cultured on PCL nanofibers showed the greatest F-actin disruption, while iPSC-CMs presented the greatest cytoskeletal disturbances for cultures on PU nanofibrous materials. Also, HCM and iPSC-CM cells differ in HIF-1α expression, which may be due to different cell physiology and their response to hypoxia and culture on 3D structures.

### Type of cell death after hypoxia and hypoxia with re-oxygenation

**Fig. 3:**
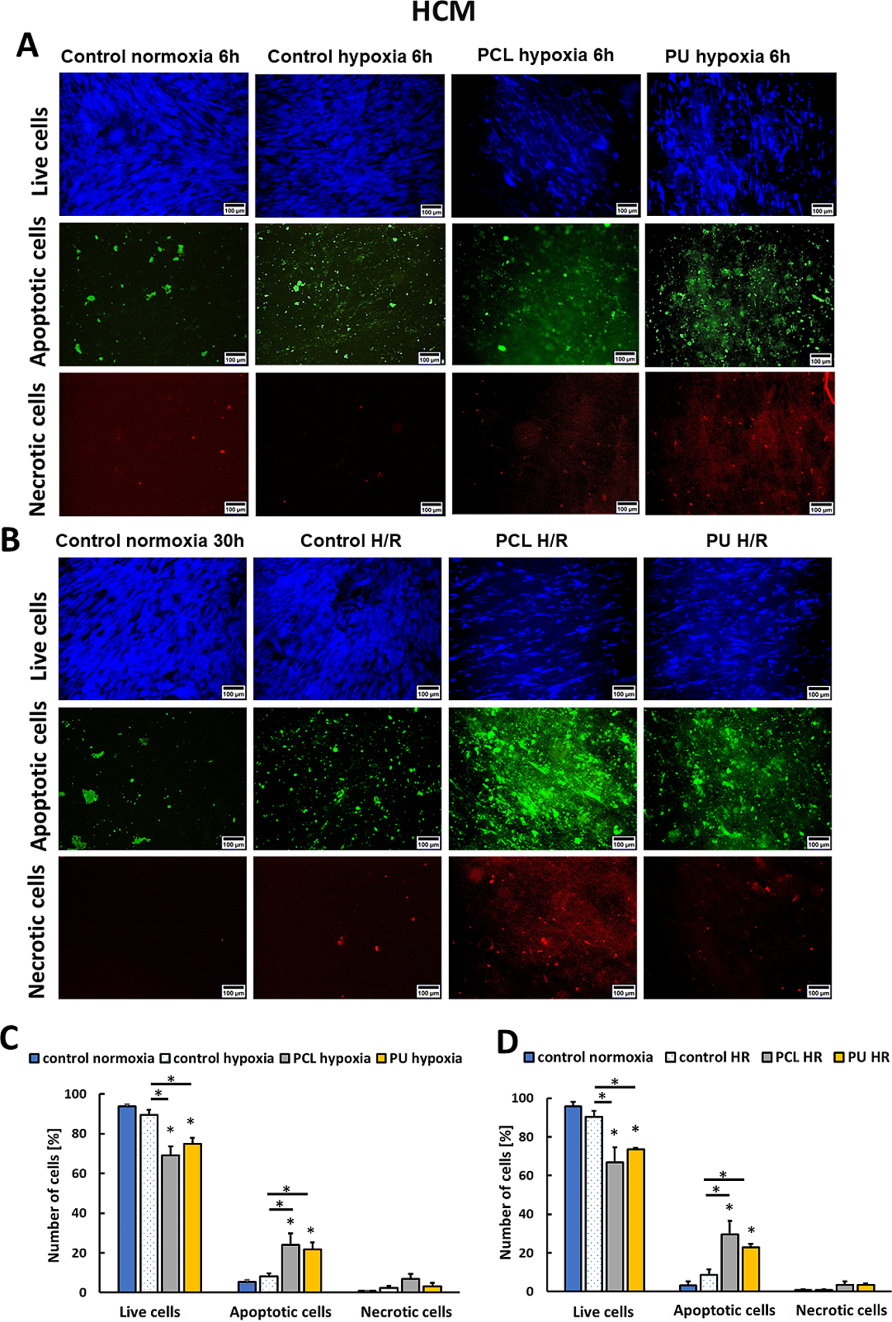
Results of cell death type determination for HCM cells after 6 h of hypoxia (**A, C**) and hypoxia with re-oxygenation (6/24 h) (**B, D**) cultured on PU and PCL nanofibrous mats and PS (hypoxia or H/R control) compared to cells cultured in normoxia. *- *p* < 0.05- statistically significant differences with control normoxia, *with line-*p* < 0.05 statistically significant differences between groups. *n* > 3. Scale bar 100 μm

Degradation of F-actin indicated that cells may begin to go into apoptosis. We noticed different HIF-1α levels depending on the culture conditions (normoxia, hypoxia, or hypoxia with re-oxygenation) and the culture substrates (PS, PCL, and PU nanofibrous mats). Hypoxia and hypoxia with re-oxygenation caused a significant increase of apoptotic cells for cultures performed on nanofibrous mats (Fig. 3). For HCM, the number of apoptotic cells was 5.3%, 8.1%, and 24.0%, 21.8% for control normoxia, control hypoxia, hypoxia on PCL, and PU nanofibrous mats, respectively (Fig. 3C). The number of apoptotic HCM cells equaled 3.2%, 8.6%, 29.6%, 23% for control normoxia, control H/R, H/R on PCL, and PU nanofibrous mats, respectively (Fig. 3D).

**Fig. 4:**
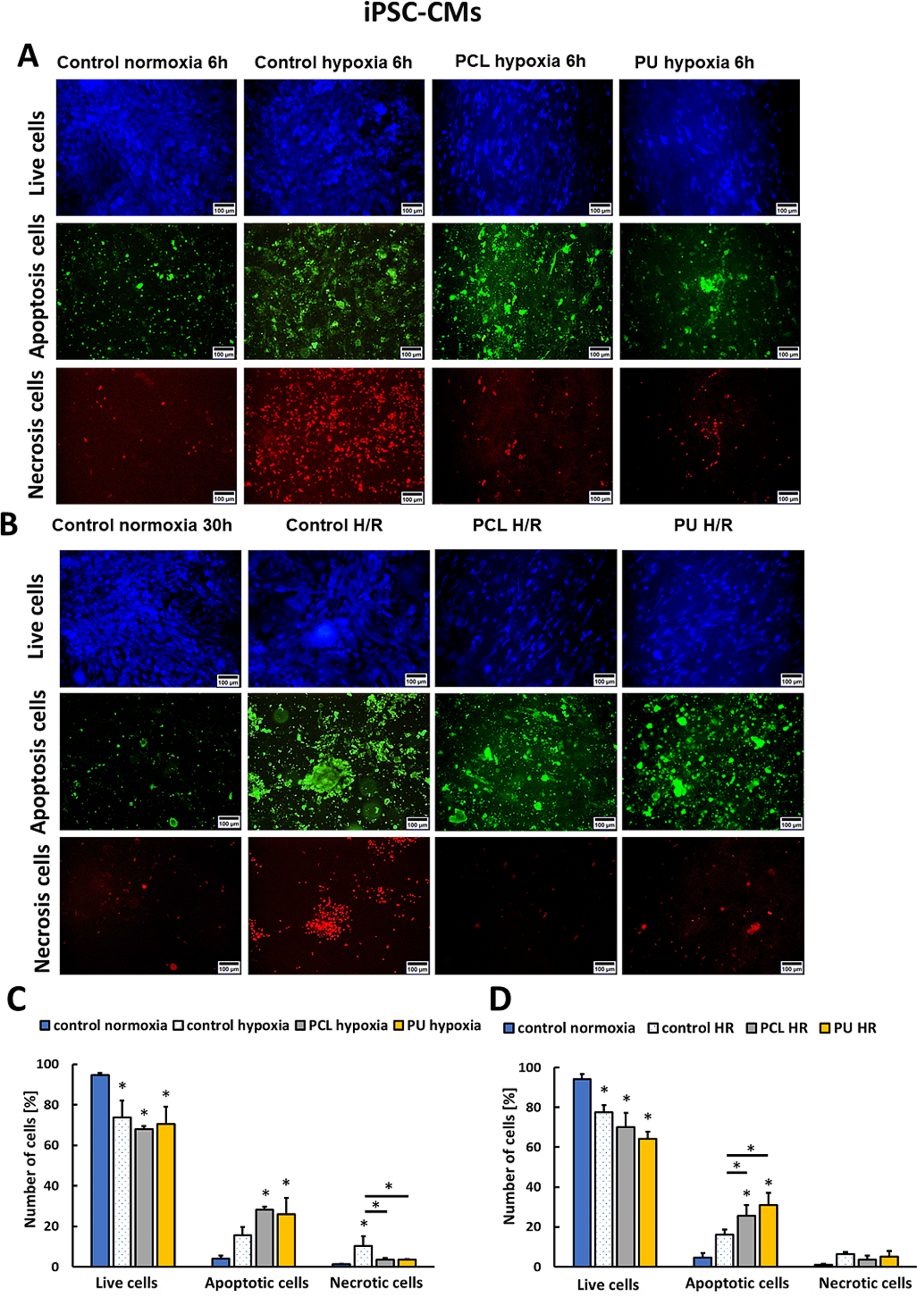
Results of cell death type determination for iPSC-CMs cells after 6 h of hypoxia (**A, C**) or hypoxia with re-oxygenation (6/24 h) (**B, D**) cultured on PU and PCL nanofibers and PS (hypoxia or H/R control) compared to cells cultured in normoxia. *- *p* < 0.05- statistically significant differences with control normoxia, *with line-*p* < 0.05 statistically significant differences between groups. *n* > 3. Scale bar 100 μm

According to the literature, iPSC-CMs are immature cells that may become less sensitive to anaerobic conditions and undergo more necrotic death during hypoxia than adult cardiomyocytes [39, 40]. iPSC-CMs cultured on PS under hypoxia caused an increase in the number of necrotic cells (10.5%) (Fig. 4A). For cultures performed on PCL and PU nanofibrous mats, it equaled 3.6%. However, we noted that iPSC-CM culture on nanofibrous mats after hypoxia and hypoxia with re-oxygenation showed a much lower percentage of necrotic cells. Culture conducted on nanofiber mats can indirectly affect the cell death pathway that the cell enters. Similarly to HCM cells, the increase of the number of apoptotic iPSC-CM cells under hypoxia was also noticed (for PS, PCL, and PU nanofibrous mats was 15.7%, 28.3%, and 25.9%, respectively) to compare with normoxia control (4.1%) (Fig. 4A and C). The number of apoptotic cells was 4.7%, 16.1%, 25.5%, and 30.1% for control normoxia, control under H/R, PCL, and PU nanofibrous mats under H/R (Fig. 4B and D). We noticed that iPSC-CM culture on nanofibrous mats after hypoxia and hypoxia with re-oxygenation induces apoptotic cell death. This is more characteristic of adult cardiomyocytes in vivo [41].

Based on the above results, HCM and iPSC-CMs cultures grown on nanofibrous mats after hypoxia and hypoxia with re-oxygenation showed a significant increase in number of apoptotic cells compared to cells cultured on polystyrene plates. Moreover, there is an increase in the number of apoptotic cells after H/R compared to cultures after hypoxia, but it is not statistically significant.

### Analysis of gene expression under hypoxia and H/R

**Fig. 5:**
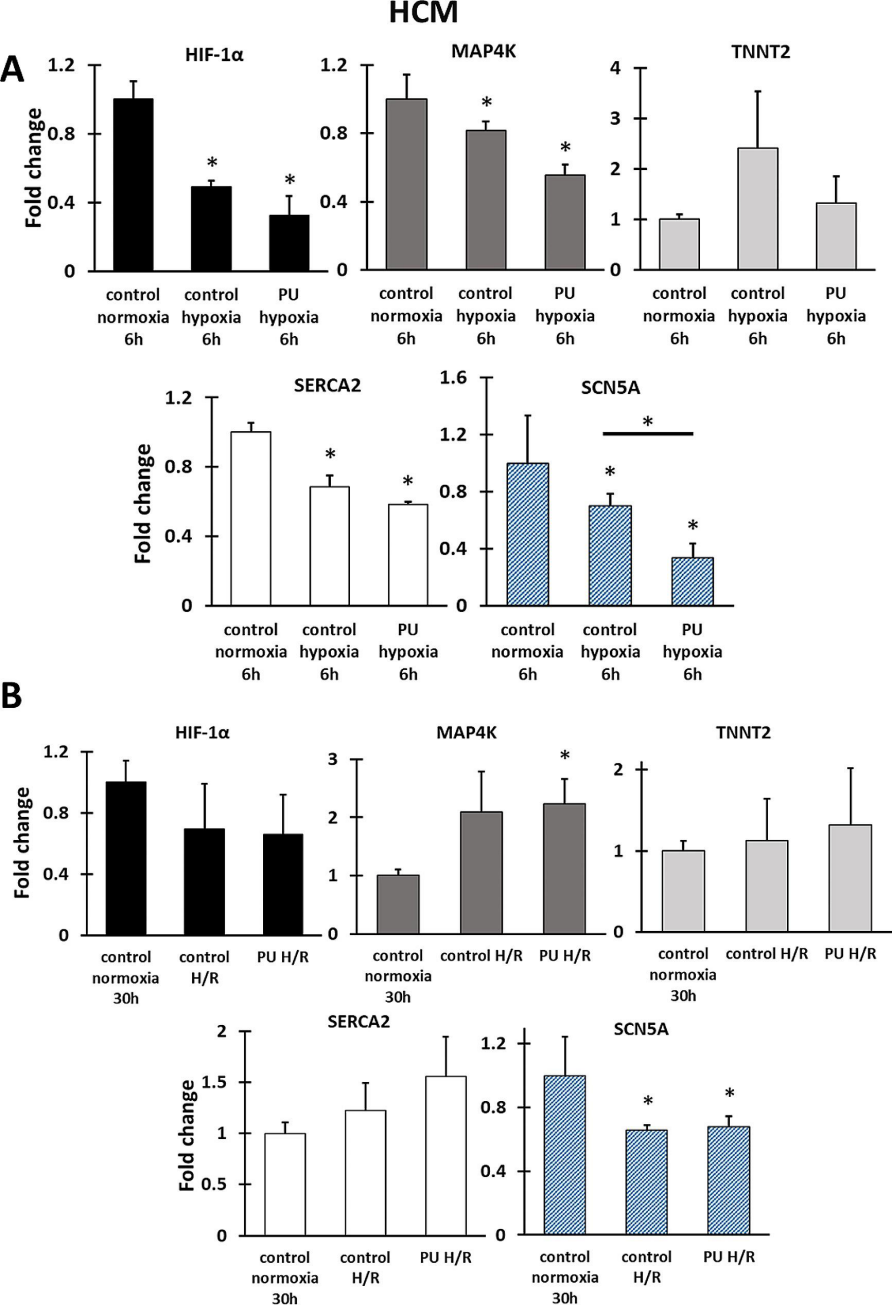
Gene expression analysis of HCM cells cultured under hypoxia (**A**) and hypoxia with re-oxygenation (**B**). HIF-1α encoding hypoxia-inducible factor 1-alpha, MAP4K encoding mitogen-activated protein kinase kinase kinase kinase, TNNT2 encoding troponin T, SERCA2 encoding calcium-ATPase type 2, and SCN5A encoding sodium channel protein type 5 subunit alpha. * - *p* < 0.05 – statistically significant differences were determined by comparison with the cells cultured on a polystyrene plate (control normoxia). *with line-*p* < 0.05 statistically significant differences between groups. *n* = 3

**Fig. 6:**
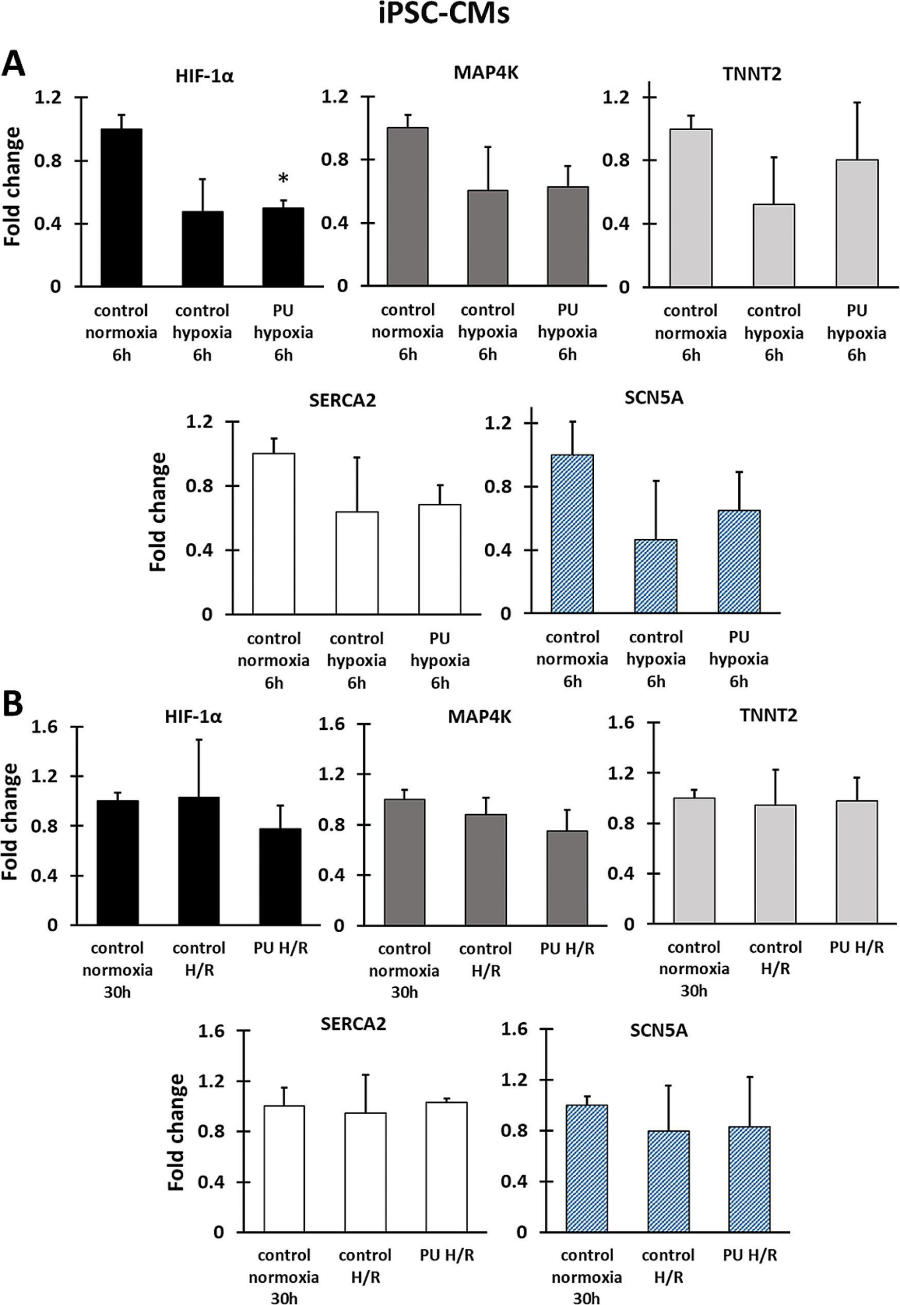
Gene expression analysis of iPSC-CMs cells cultured under hypoxia (**A**) and hypoxia with reoxygenation (**B**). HIF-1α encoding hypoxia-inducible factor 1-alpha, MAP4K encoding mitogen-activated protein kinase kinase kinase kinase, TNNT2 encoding troponin T, SERCA2 encoding calcium-ATPase type 2, and SCN5A encoding sodium channel protein type 5 subunit alpha. * - *p* < 0.05 – statistically significant differences were determined by comparison with the cells cultured on a polystyrene plate (control normoxia). *n* = 3

The changes in gene expression under hypoxia and H/R by RT-PCR analysis were carried out for five genes (Figs. 5 and 6): hypoxia-inducible factor 1-alpha (*HIF-1α*), mitogen-activated protein kinase kinase kinase kinase (*MAP4K*), Troponin T (*TNNT2*), calcium-ATPase type 2 (*SERCA2*), and sodium voltage-gated channel alpha subunit 5 (*SCN5A*). The genes studied were related to cell changes under hypoxia and disorders that may indicate cell dysfunction or damage. Changes in *HIF-1α* expression are a response of cardiomyocytes to hypoxia and are involved in the triggering of a number of pathophysiological processes [42]; *MAP4K* is responsible for the regulation of oxidative stress, induction of inflammation and cell death [43], *TNNT2* regulates cardiomyocytes function [44], changes in the expression of genes encoding ion pumps (such as *SCN5A* and *SERCA2*) may indicate disturbances in membrane potential [45, 46]. For gene expression analysis, PU nanofibrous mats were selected. Performing RT-PCR for cells cultured on PCL nanofiber mats was difficult due to the limited amount of RNA extracted for the study.

For HCM cells, the expression of most genes decreased (*HIF-1α, MAP4K, SERCA2, SCN5A*) under hypoxia conditions (Fig. 4A). For all tested genes, the greater decrease in level of gene expression for cultures performed on PU nanofibrous mats than on a polystyrene plate were determined. However, only for *SCN5A was it* statistically significant. The level of the *TNNT2* expression increased for both types of cultures under hypoxia (2.4-fold for PS and 1.3-fold for PU nanofibrous mats compared to normoxia control). For HCM cells maintained under hypoxia with re-oxygenation, the level of *TNNT2* expression is higher than in controls; however, it is lower compared to cultures under hypoxia. Additionally, there is an increase in the level of *MAP4K* expression for cells cultured under H/R, which may indicate that re-oxygenation causes increased oxidative stress and triggers inflammation. Also, the expression of *SERCA2* increased, and it is higher for cells grown on PU nanofibrous mats. The expression of the other genes (*HIF-1a* and *SCN5A*) decreased for H/R compared to the control normoxia.

For iPSC-CMs, the changes in gene expressions were not significant. Figure 6A shows that the expression level of all selected genes decreases under hypoxia for cultures on nanofibrous mats and polystyrene plates compared to normoxia controls. For the *HIF-1α*, a significant difference in expression was noticed between cells cultured on PU nanofibrous mats and control normoxia. For the other selected genes, the changes in expression are statistically insignificant when cultures on PU nanofibrous mats are compared to normoxia and hypoxia controls. Based on the results in Fig. 6B, the expression of all study genes of cultures grown on PU nanofibrous mats and PS after hypoxia with re-oxygenation increases compared to the expression for cells under hypoxia. The expression level of genes for the test cultures (iPSC after H/R) is similar to that of the normoxia control. These results may indicate that iPSC-CMs cells are less sensitive to hypoxia and that after receiving oxygen again, cellular repair mechanisms restore cells to their pre-hypoxic state in both 2D and 3D cultures.

## Discussion

Nanofibrous mats form three-dimensional (3D) porous, gas-permeable structures, have a large surface-to-volume ratio, and can be used as culture substrates, which imitate the extracellular matrix of cardiac tissue [47]. Additionally, they have been found as materials that affect the morphology and physiology of cardiac cells. It was noticed that the cells have a more elongated, rod-like shape and could maturate [17, 23, 48]. The cells grown on nanofibers are morphologically and functionally more similar to adult cardiomyocytes than those cultured on polystyrene plates [22–24, 28]. Based on the literature, it is determined that cardiomyocytes cultured on nanofibers more accurately reflect the function of adult cardiac tissue than 2D cultures [48–50]. Therefore, it is worth testing whether nanofibrous mats will also affect the response of human cardiomyocytes to hypoxia and hypoxia with re-oxygenation. Hypoxia is a major cause of myocardial damage in cardiovascular diseases [1]; however, more and more research has indicated that the influence on damaged cardiac cells has also delivered oxygen back to CMs after hypoxia [7–10]. Therefore, studying the cellular response to hypoxia and H/R on a substrate that mimics in vivo conditions is necessary. To the best of our knowledge, there are no studies on the effects of hypoxia on cardiomyocyte cells cultured on nanofibrous mats. Due to the specific properties of nanofibrous mats, perhaps using them as a substrate for cells under hypoxia will allow for a cellular response that more closely resembles their response in vivo. Due to the limited number of human cardiac in vitro models, verifying how not only human cardiomyocytes but also cardiomyocytes derived from induced pluripotent stem cells cultured on nanofibrous mats with a 3D structure would behave under hypoxia and hypoxia with re-oxygenation was determined.

HCM cells are primary human cardiomyocytes, similar to mature cardiomyocytes in morphology and physiology. In addition, the metabolism of these cells is based on fatty acid oxidation rather than glycolysis, as in fetal cells [51]. However, they do not contract, and their usage is limited due to the number of passages that can be made [52] (see Supplementary materials). iPSC-CMs are the cells that are increasingly used in regenerative medicine despite their differences from mature cardiomyocytes. iPSCs, which differentiate into cardiomyocytes, can be derived from a selected patient; therefore, using iPSC-CMs provides a chance for individual analysis of the condition. This is important because the phenotype of the same disease can vary from one organism to another. Therefore, using iPSC-CMs to develop a model of a patient’s heart tissue may allow the cause of a specific disorder to be understood Therefore, the use of iPSC-CMs to develop a model of a patient’s cardiac tissue can allow the treatment of not only the symptoms but also the cause of the onset of a particular disorder [53]. Additionally, more and more research is being done on using induced pluripotent stem cells to generate cardiac organoids [54, 55]. An organoid is a 3D multicellular structure formed through a self-organization [55]. Cardiac organoids physiologically mimic the development of immature cardiac tissue [54]; therefore, in further studies, they may be a suitable in vitro model for the human heart. They may also be useful for mimicking human heart tissue under hypoxic or H/R conditions.

For both HCM and iPSC-CMs cells under hypoxia, F-actin filaments are disrupted for cultures on nanofibrous mats and polystyrene plates. However, this disruption is maintained only for cultures on nanofibrous mats after H/R. According to the literature, the disrupted organization of the cytoskeletal structure of adult cardiomyocytes after hypoxia and H/R indicates cell damage [56]. The HIF-1α increase is due to damage and the occurrence of cellular stress [52], which we also observed in HCM cultures on nanofibrous mats. For iPSC-CMs, the decrease in the level of HIF-1α protein for cultures under hypoxia was noticed. However, the level of HIF-1α in the cells grown on nanofibrous materials after H/R is higher than in cultures after hypoxia. In the literature, the decrease in HIF-1α is explained that its level is regulated by oxygen-independent pathways [57]. Based on the literature, mechanical stress can influence the decrease of HIF-1α [40]. Mechanical stress can be affected by the elasticity of the substrate on which cardiomyocytes are cultured [58]. In our study, nanofibrous mats differing in elasticity were used. From the results, PU nanofibers with lower elasticity cause a greater decrease in HIF-1α than PCL nanofibers. In our research, it is noticed that HIF-1α protein expression differs from the gene expression. This may be due to the *HIF-1α* gene increasing under the influence of hypoxia and dropping rapidly when cells come into contact with oxygen [59]. The level of HIF-1α protein is stabilized longer [60], which made it possible to observe it during immunostaining. *HIF-1α* mRNA is degraded very rapidly when exposed to oxygen [59].

One of the cellular responses to hypoxia observed in cardiac tissue is apoptosis of cardiomyocytes [61]. Our work showed a significant increase in apoptotic cells for HCMs and iPSC-CMs cultured on nanofibrous mats under hypoxia or H/R compared to cultures on PS. Moreover, there was a slight increase in the number of damaged cells after H/R than after hypoxia for iPSC-CM and HCM cells cultured on nanofibrous mats. The decrease in cardiomyocyte viability after H/R is also observed in other studies with iPSC-CMs. However, the study used a standard culture surface [26]. Re-oxygenation results in the supply of oxygen to the cells again and the restoration of pre-hypoxia conditions, but it can trigger biological mechanisms that cause cardiac cell dysfunction [62]. Similarly, in the above studies, HCM after H/R shows an increase in *MAP4K* gene expression, which is an indicator of inflammation in the cell [63]. For HCM, a higher level of *TNNT2* expression was determined under hypoxia than for control normoxia and cultures after H/R. In contrast, expression *TNNT2* in iPSC-CMs is downregulated. *TNNT2* encodes cardiac troponin T, which regulates the function of cardiomyocytes under hypoxia. Based on the literature, fetal cardiomyocytes mainly adopted anaerobic glycolysis under hypoxia, which induces reduced expression of cardiac troponin T. In contrast, adult cardiac cells used oxidative phosphorylation as energy suppliers and upregulated cardiac troponin T in hypoxia conditions [64]. Our research shows no significant differences between 2D and 3D cultures. Additionally, after H/R, the expression of this gene was similar in hypoxia cultures and normoxia controls. The decreased expression of *SCN5A* and *SERCA2* was also noticed for cultures of HCM and iPSC-CMs under hypoxia. Moreover, these genes’ levels are lower for HCM grown on PU nanofibrous mats than for cultures on PS. In contrast, HCM and iPSC-CMs under H/R gave different cellular responses. The level of these genes for iPSC-CMs under H/R is restored to a pre-hypoxic state in both 2D and 3D cultures. The expression of *SCN5A* for HCM cultures after H/R is lower than for normoxia, whereby the lowest for cultures on nanofibers. In turn, *SERCA2* expression increases and is highest for cultures for nanofibrous mats. As a result of cardiac hypoxia, metabolic pathways are activated, which reduce the oxygen demand and protect cardiomyocytes from the negative effects of its lack [65]. The anaerobic condition reduces pyruvate dehydrogenase activity, which in turn causes the conversion of pyruvate to lactate [26]. The accumulation of lactate involves acidification of the cellular environment, which leads to disturbances in the binding of calcium to troponin. Reperfusion is necessary for cell survival but also causes changes that can negatively affect cardiomyocyte functioning. Such changes include accumulation in cardiac cells Na^+^ instead of H^+^, which activates the pumps responsible for exchanging Na^+^ ions into Ca^2+^. Changes in ion transport also result in disturbances in membrane potential. This can induce a decrease in the level of *SCN5A* expression [45]. The expression level of the *SERCA2* gene, encoding calcium ATPase, responsible for the transfer of calcium ions from the cytosol to the sarcoplasmic reticulum, decreases during hypoxia in the heart (also causing Ca^2+^ accumulation) while restoring the availability of oxygen to cells and increases the expression of this gene to prevent the development of contractile dysfunction [46]. Based on the above data, two in vitro models were obtained in which the functioning of two types of human cardiomyocytes was compared in 3D and 2D models, which allowed us to study the response of the cells to hypoxia and hypoxia with reoxygenation.

## Conclusion

In vivo conditions, human cardiac tissue is formed by fibers composed of cardiomyocytes that form a 3D structure supported by extracellular matrix proteins. Nanofibrous mats can imitate the heart ECM structurally and functionally in 3D cardiac in vitro models. In the present study, we analyzed the type of cell death, expression of selected genes, and proteins in which the changes occur in cardiomyocytes cultured on nanofibrous mats under hypoxia and hypoxia with re-oxygenation. The research utilized primary human cardiomyocytes (HCM) and human pluripotent stem cell-derived cardiomyocytes (iPSC-CMs) cultured on polystyrene plate (PS), poly(ε-caprolactone) (PCL) nanofibers and polyurethane (PU) nanofibers. It was noticed that nanofibrous mats affect the cellular response of HCM and iPSC-CM to hypoxia and hypoxia with re-oxygenation. Primary human cardiomyocytes cultured on nanofibrous mats significantly increased HIF-1α protein levels and the number of damaged cells after hypoxia. Also, these changes are sustained after re-oxygenation. In contrast, such significant changes are not determined for iPSC-CM cells. However, using iPSC-CMs in 3D models has potential, and further studies are required. These studies can support research to find in vitro cardiac models to accurately imitate human cardiac tissue during ischemia or ischemia with reperfusion. This will enable effective research to find a treatment for damaged human cardiac tissue.

### Electronic supplementary material

Below is the link to the electronic supplementary material.

Supplementary Material 1

## Data Availability

The datasets generated during the current study are available from the corresponding author upon reasonable request.

## References

[1] Stein JM, Mummery CL, Bellin M. Engineered models of the human heart: directions and challenges. Stem Cell Rep. Sep. 2021;16(9):2049–57. 10.1016/j.stemcr.2020.11.013. 10.1016/j.stemcr.2020.11.013 PMC8452488 33338434

[2] Wang Y et al. Sep., ‘Fibroblasts in heart scar tissue directly regulate cardiac excitability and arrhythmogenesis’, Science. 2023;381(6665):1480–1487. 10.1126/science.adh9925. 10.1126/science.adh9925 PMC10768850 37769108

[3] Ashtari K et al. Apr., ‘Electrically conductive nanomaterials for cardiac tissue engineering’, Advanced Drug Delivery Reviews. 2019;144:162–179. 10.1016/j.addr.2019.06.001. 10.1016/j.addr.2019.06.001 PMC6784829 31176755

[4] Brzozka Z, Jastrzebska E (2018). Cardiac Cell Culture technologies.

[5] Alharbi KS, et al. A narrative review on the biology of piezo1 with platelet-rich plasma in cardiac cell regeneration. Chemico-Biol Interact. Aug. 2022;363:110011. 10.1016/j.cbi.2022.110011. 10.1016/j.cbi.2022.110011 35728671

[6] Lucero García Rojas EY, Villanueva C, Bond RA. Hypoxia inducible factors as Central players in the Pathogenesis and Pathophysiology of Cardiovascular diseases. Front Cardiovasc Med. Aug. 2021;8:709509. 10.3389/fcvm.2021.709509. 10.3389/fcvm.2021.709509 PMC8382733 34447792

[7] Tan C, Li J, Yuan Z, Mu Y. ‘Circular RNA ciRs-126 promotes hypoxia/reoxygenation cardiac injury possibly through miR-21’, Thrombosis J. Dec. 2022;20(1):2. 10.1186/s12959-021-00355-x. 10.1186/s12959-021-00355-x PMC8725357 34983563

[8] Ali SS, Noordin L, Bakar RA, Zainalabidin S, Jubri Z. and W. A. N. Wan Ahmad, ‘Current Updates on Potential Role of Flavonoids in Hypoxia/Reoxygenation Cardiac Injury Model’, Cardiovasc Toxicol. Aug. 2021;21(8):605–618. 10.1007/s12012-021-09666-x. 10.1007/s12012-021-09666-x 34114196

[9] Xu J, Huang J, He X, Hu M, Su S, Liu P. ‘Myosin 1b Participated in the Modulation of Hypoxia/Reoxygenation-Caused H9c2 Cell Apoptosis and Autophagy’. Analytical Cellular Pathology. Nov. 2022;2022:1–10. 10.1155/2022/5187304. 10.1155/2022/5187304 PMC9708368 36458211

[10] Niu R, et al. MicroRNA-582‐5p targeting Creb1 modulates apoptosis in cardiomyocytes hypoxia/reperfusion‐induced injury. Immunity Inflam Disease. Nov. 2022;10(11):e708. 10.1002/iid3.708. 10.1002/iid3.708 PMC9601879 36301033

[11] Wu D, Yotnda P. ‘Induction and Testing of Hypoxia in Cell Culture’. JoVE. Aug. 2011;54:2899. 10.3791/2899. 10.3791/2899 PMC3217626 21860378

[12] Wu J-W, Hu H, Hua J, Ma L-K (2022). ATPase inhibitory factor 1 protects the heart from acute myocardial ischemia/reperfusion injury through activating AMPK signaling pathway. Int J Biol Sci.

[13] Xing Y, et al. Blunting TRPML1 channels protects myocardial ischemia/reperfusion injury by restoring impaired cardiomyocyte autophagy. Basic Res Cardiol. Dec. 2022;117(1). 10.1007/s00395-022-00930-x. 10.1007/s00395-022-00930-x 35389129

[14] Xu L, Chen R, Ma X, Zhu Y, Sun G, Sun X. ‘Scutellarin protects against myocardial ischemia-reperfusion injury by suppressing NLRP3 inflammasome activation’. Phytomedicine. Mar. 2020;68: 153169. 10.1016/j.phymed.2020.153169. 10.1016/j.phymed.2020.153169 31999976

[15] Tang L-J et al. Jan., ‘Ubiquitin-specific protease 7 promotes ferroptosis via activation of the p53/TfR1 pathway in the rat hearts after ischemia/reperfusion’. Free Radical Biology and Medicine. 2021;162:339–352. 10.1016/j.freeradbiomed.2020.10.307. 10.1016/j.freeradbiomed.2020.10.307 33157209

[16] Berthiaume F, Maguire TJ, Yarmush ML. ‘Tissue Engineering and Regenerative Medicine: History, Progress, and Challenges’, Annu. Rev. Chem. Biomol. Eng. Jul. 2011;2(1):403–430. 10.1146/annurev-chembioeng-061010-114257. 10.1146/annurev-chembioeng-061010-114257 22432625

[17] Cristallini C, Vitale E, Giachino C, Rastaldo R. ‘Nanoengineering in Cardiac Regeneration: Looking Back and Going Forward’, Nanomaterials. Aug. 2020;10(8):1587. 10.3390/nano10081587. 10.3390/nano10081587 PMC7466652 32806691

[18] Wan ACA, Ying JY. Nanomaterials for in situ cell delivery and tissue regeneration☆. Adv Drug Deliv Rev. Jun. 2010;62:7–8. 10.1016/j.addr.2010.02.002. 10.1016/j.addr.2010.02.002 20156499

[19] Mohammadi Nasr S et al. Jun., ‘Biodegradable Nanopolymers in Cardiac Tissue Engineering: From Concept Towards Nanomedicine’, IJN. 2020;15:4205–4224. 10.2147/IJN.S245936. 10.2147/IJN.S245936 PMC7314574 32606673

[20] Tomecka E, Wojasinski M, Jastrzebska E, Chudy M, Ciach T, Brzozka Z. ‘Poly(l -lactic acid) and polyurethane nanofibers fabricated by solution blow spinning as potential substrates for cardiac cell culture’, Materials Science and Engineering: C. Jun. 2017;75:305–316. 10.1016/j.msec.2017.02.055. 10.1016/j.msec.2017.02.055 28415467

[21] ‘Solution blow spun poly-L-lactic acid/ceramic fibrous composites for bone implant applications’, Chemical and Process Engineering. Nov. 2023, 10.24425/cpe.2021.138931.

[22] Karimi SNH, Aghdam RM, Ebrahimi SAS, Chehrehsaz Y. ‘Tri-layered alginate/poly(ε caprolactone) electrospun scaffold for cardiac tissue engineering’, Polymer International. Sep. 2022;71(9):1099–1108. 10.1002/pi.6371.

[23] Zhang M, et al. Three-dimensional Poly-(ε-Caprolactone) nanofibrous scaffolds promote the maturation of human pluripotent stem cells-Induced cardiomyocytes. Front Cell Dev Biol. Aug. 2022;10:875278. 10.3389/fcell.2022.875278. 10.3389/fcell.2022.875278 PMC9377449 35979378

[24] Najafi Tireh Shabankareh A, Samadi Pakchin P, Hasany M, Ghanbari H. Development of a new electroconductive nanofibrous cardiac patch based on polyurethane-reduced graphene oxide nanocomposite scaffolds. Mater Chem Phys. Sep. 2023;305:127961. 10.1016/j.matchemphys.2023.127961.

[25] Yang H et al. Jul., ‘Transcriptome analysis of non human primate-induced pluripotent stem cell-derived cardiomyocytes in 2D monolayer culture vs. 3D engineered heart tissue’. Cardiovascular Research. 2021;117(9):2125–2136. 10.1093/cvr/cvaa281. 10.1093/cvr/cvaa281 PMC8318103 33002105

[26] Veldhuizen J et al. Feb., ‘Cardiac ischemia on-a-chip to investigate cellular and molecular response of myocardial tissue under hypoxia’. Biomaterials. 2022;281:121336. 10.1016/j.biomaterials.2021.121336. 10.1016/j.biomaterials.2021.121336 PMC10440189 35026670

[27] Lutter G et al. Jan., ‘Biodegradable Poly-ε-Caprolactone Scaffolds with ECFCs and iMSCs for Tissue-Engineered Heart Valves’, IJMS. 2022;23(1):527. 10.3390/ijms23010527. 10.3390/ijms23010527 PMC8745109 35008953

[28] Kołodziejek D, Łopianiak I, Tadko O, Drozd M, Wojasiński M, Jastrzębska E. Magnetic polyurethane nanomaterials: a novel approach for in vitro cardiac cell maturation and culture. Polym Test. Oct. 2023;127:108190. 10.1016/j.polymertesting.2023.108190.

[29] Fakhrali A et al. Oct., ‘Biocompatible graphene-embedded PCL / PGS ‐based nanofibrous scaffolds: A potential application for cardiac tissue regeneration’. J of Applied Polymer Sci. 2021;138(40):51177. 10.1002/app.51177.

[30] Ahmadi P, Nazeri N, Derakhshan MA, Ghanbari H. ‘Preparation and characterization of polyurethane/chitosan/CNT nanofibrous scaffold for cardiac tissue engineering’, International Journal of Biological Macromolecules. Jun. 2021;180:590–598. 10.1016/j.ijbiomac.2021.03.001. 10.1016/j.ijbiomac.2021.03.001 33711373

[31] Homaeigohar S, Boccaccini AR. Nature-derived and synthetic additives to poly(ɛ-Caprolactone) Nanofibrous Systems for Biomedicine; an updated overview. Front Chem. Jan. 2022;9:809676. 10.3389/fchem.2021.809676. 10.3389/fchem.2021.809676 PMC8807494 35127651

[32] Jirofti N, Mohebbi-Kalhori D, Samimi A, Hadjizadeh A, Kazemzadeh GH. Small-diameter vascular graft using co-electrospun composite PCL/PU nanofibers. Biomed Mater. Aug. 2018;13(5):055014. 10.1088/1748-605X/aad4b5. 10.1088/1748-605X/aad4b5 30026407

[33] Ward MC, Gilad Y. ‘A generally conserved response to hypoxia in iPSC-derived cardiomyocytes from humans and chimpanzees’, eLife. Apr. 2019;8:e42374. 10.7554/eLife.42374. 10.7554/eLife.42374 PMC6538380 30958265

[34] Iwoń Z, et al. Improving rodents and humans cardiac cell maturity in vitro through polycaprolactone and polyurethane nanofibers. Biomed Mater. Mar. 2024;19(2):025031. 10.1088/1748-605X/ad240a. 10.1088/1748-605X/ad240a 38290152

[35] Liszewska E, et al. Establishment of two hiPSC lines (IIMCBi001-A and IIMCBi002-A) from dermal fibroblasts of healthy donors and characterization of their cell cycle. Stem Cell Res. Apr. 2021;52:102225. 10.1016/j.scr.2021.102225. 10.1016/j.scr.2021.102225 33588215

[36] Lian X et al. Jan., ‘Directed cardiomyocyte differentiation from human pluripotent stem cells by modulating Wnt/β-catenin signaling under fully defined conditions’, Nat Protoc. 2013;8(1):162–175. 10.1038/nprot.2012.150. 10.1038/nprot.2012.150 PMC3612968 23257984

[37] Kołodziejek D, et al. Cardiac tissue modeling using Flow microsystems and Nanofiber mats: evaluating Hypoxia-Induced Cellular and Molecular Changes. Sens Actuators B. p. Dec. 2023;135169. 10.1016/j.snb.2023.135169.

[38] Peters MC et al. Oct., ‘Metabolic Maturation Increases Susceptibility to Hypoxia-induced Damage in Human iPSC-derived Cardiomyocytes’, Stem Cells Translational Medicine. 2022;11(10):1040–1051. 10.1093/stcltm/szac061. 10.1093/stcltm/szac061 PMC9585948 36018047

[39] Häkli M, et al. Human induced pluripotent stem cell-based platform for modeling cardiac ischemia. Sci Rep. Feb. 2021;11(1):4153. 10.1038/s41598-021-83740-w. 10.1038/s41598-021-83740-w PMC7893031 33603154

[40] Häkli M et al. ‘Electrophysiological Changes of Human-Induced Pluripotent Stem Cell-Derived Cardiomyocytes during Acute Hypoxia and Reoxygenation’, Stem Cells International. Dec. 2022;2022:1–15. 10.1155/2022/9438281. 10.1155/2022/9438281 PMC9792238 36579142

[41] Ouyang M, Lu J, Ding Q, Qin T, Peng C, Guo Q. ‘Knockdown of long non-coding RNA PVT1 protects human AC16 cardiomyocytes from hypoxia/reoxygenation-induced apoptosis and autophagy by regulating miR-186/Beclin-1 axis’, Gene. Sep. 2020;754:144775. 10.1016/j.gene.2020.144775. 10.1016/j.gene.2020.144775 32428696

[42] Elgenaidi IS, Spiers JP. ‘Hypoxia modulates protein phosphatase 2A through HIF-1α dependent and independent mechanisms in human aortic smooth muscle cells and ventricular cardiomyocytes’, British J Pharmacology. Jun. 2019;176(11):1745–1763. 10.1111/bph.14648. 10.1111/bph.14648 PMC6514298 30825189

[43] Quaglio AEV, Castilho ACS, Di Stasi LC. ‘Experimental evidence of MAP kinase gene expression on the response of intestinal anti-inflammatory drugs’, Life Sciences. Sep. 2015;136:60–66. 10.1016/j.lfs.2015.06.012. 10.1016/j.lfs.2015.06.012 26141991

[44] Liu Y, et al. Cardiac troponin T (TNNT2) plays a potential oncogenic role in colorectal carcinogenesis. Cancer Cell Int. Jul. 2023;23(1):146. 10.1186/s12935-023-02977-9. 10.1186/s12935-023-02977-9 PMC10363310 37481519

[45] Scuderi GJ, Butcher J. Naturally Engineered Maturation of Cardiomyocytes. Front Cell Dev Biol. May 2017;5:50. 10.3389/fcell.2017.00050. 10.3389/fcell.2017.00050 PMC5418234 28529939

[46] Gonnot F, et al. SERCA2 phosphorylation at serine 663 is a key regulator of Ca2 + homeostasis in heart diseases. Nat Commun. Jun. 2023;14(1):3346. 10.1038/s41467-023-39027-x. 10.1038/s41467-023-39027-x PMC10250397 37291092

[47] Mousa HM, et al. Development of biocompatible tri-layered nanofibers patches with endothelial cells for cardiac tissue engineering. Eur Polymer J. Apr. 2020;129:109630. 10.1016/j.eurpolymj.2020.109630.

[48] Ding M, et al. Aligned nanofiber scaffolds improve functionality of cardiomyocytes differentiated from human induced pluripotent stem cell-derived cardiac progenitor cells. Sci Rep. Aug. 2020;10(1):13575. 10.1038/s41598-020-70547-4. 10.1038/s41598-020-70547-4 PMC7419298 32782331

[49] Tian F et al. Apr., ‘Aligned Nanofibrous Net Deposited Perpendicularly on Microridges Supports Endothelium Formation and Promotes the Structural Maturation of hiPSC-Derived Cardiomyocytes’, ACS Appl. Mater. Interfaces. 2023;15(14):17518–17531. 10.1021/acsami.2c22551. 10.1021/acsami.2c22551 36992621

[50] Khan M, et al. Evaluation of changes in morphology and function of Human Induced Pluripotent Stem Cell Derived cardiomyocytes (HiPSC-CMs) cultured on an aligned-Nanofiber Cardiac Patch. PLoS ONE. May 2015;10(5):e0126338. 10.1371/journal.pone.0126338. 10.1371/journal.pone.0126338 PMC4437999 25993466

[51] Karbassi E, et al. Cardiomyocyte maturation: advances in knowledge and implications for regenerative medicine. Nat Rev Cardiol. Jun. 2020;17(6):341–59. 10.1038/s41569-019-0331-x. 10.1038/s41569-019-0331-x PMC7239749 32015528

[52] Hafez P et al. Sep., ‘Development of an In Vitro Cardiac Ischemic Model Using Primary Human Cardiomyocytes’, Cardiovasc Eng Tech. 2018;9(3):529–538. 10.1007/s13239-018-0368-8. 10.1007/s13239-018-0368-8 29948837

[53] Bizy A, Klos M. ‘Optimizing the Use of iPSC-CMs for Cardiac Regeneration in Animal Models’, Animals. Sep. 2020;10(9):1561. 10.3390/ani10091561. 10.3390/ani10091561 PMC7552322 32887495

[54] Richards DJ, et al. Human cardiac organoids for the modelling of myocardial infarction and drug cardiotoxicity. Nat Biomed Eng. Apr. 2020;4(4):446–62. 10.1038/s41551-020-0539-4. 10.1038/s41551-020-0539-4 PMC7422941 32284552

[55] Lewis-Israeli YR, et al. Self-assembling human heart organoids for the modeling of cardiac development and congenital heart disease. Nat Commun. Aug. 2021;12(1):5142. 10.1038/s41467-021-25329-5. 10.1038/s41467-021-25329-5 PMC8390749 34446706

[56] Vogler M, et al. Hypoxia modulates Fibroblastic Architecture, Adhesion and Migration: a role for HIF-1α in cofilin regulation and cytoplasmic actin distribution. PLoS ONE. Jul. 2013;8(7):e69128. 10.1371/journal.pone.0069128. 10.1371/journal.pone.0069128 PMC3715466 23874890

[57] Iommarini L, Porcelli AM, Gasparre G, Kurelac I. Non-canonical mechanisms regulating hypoxia-inducible factor 1 alpha in Cancer. Front Oncol. Nov. 2017;7:286. 10.3389/fonc.2017.00286. 10.3389/fonc.2017.00286 PMC5711814 29230384

[58] Janmey PA, Fletcher DA, Reinhart-King CA. ‘Stiffness Sensing by Cells’, Physiological Reviews. Apr. 2020;100(2):695–724. 10.1152/physrev.00013.2019. 10.1152/physrev.00013.2019 PMC7276923 31751165

[59] Liu H et al. Apr., ‘Heart-on-a-Chip Model with Integrated Extra- and Intracellular Bioelectronics for Monitoring Cardiac Electrophysiology under Acute Hypoxia’, Nano Lett. 2020;20(4):2585–2593. 10.1021/acs.nanolett.0c00076. 10.1021/acs.nanolett.0c00076 32092276

[60] Marxsen JH et al. Aug., ‘Hypoxia-inducible factor-1 (HIF-1) promotes its degradation by induction of HIF-α-prolyl-4-hydroxylases’, Biochemical Journal. 2004;381(3):761–767. 10.1042/BJ20040620. 10.1042/BJ20040620 PMC1133886 15104534

[61] Johnson TK et al. Oct., ‘Exosomes derived from induced vascular progenitor cells promote angiogenesis in vitro and in an in vivo rat hindlimb ischemia model’, American Journal of Physiology-Heart and Circulatory Physiology. 2019;317(4):H765–H776. 10.1152/ajpheart.00247.2019. 10.1152/ajpheart.00247.2019 PMC6843021 31418583

[62] Bishopric NH et al. Aug., ‘Hypoxia-activated apoptosis of cardiac myocytes requires reoxygenation or a pH shift and is independent of p53’, J. Clin. Invest. 1999;104(3):239–252. 10.1172/JCI5871. 10.1172/JCI5871 PMC408414 10430605

[63] Piacentini L, Karliner JS. ‘Altered gene expression during hypoxia and reoxygenation of the heart’, Pharmacology & Therapeutics. Jul. 1999;83(1):21–37. 10.1016/S0163-7258(99)00010-8. 10.1016/s0163-7258(99)00010-8 10501593

[64] Sun Y, et al. Effects of hypoxia on cardiomyocyte proliferation and association with stage of development. Biomedicine Pharmacotherapy. Oct. 2019;118:109391. 10.1016/j.biopha.2019.109391. 10.1016/j.biopha.2019.109391 31545287

[65] Su Z, Liu Y, Zhang H. Adaptive Cardiac Metabolism under Chronic Hypoxia: mechanism and clinical implications. Front Cell Dev Biol. Feb. 2021;9:625524. 10.3389/fcell.2021.625524. 10.3389/fcell.2021.625524 PMC7884626 33604337

