# Supplementary material for: Hypoxia and re-oxygenation effects on human cardiomyocytes cultured on polycaprolactone and polyurethane nanofibrous mats
Source: J Biol Eng. 2024 Jun 6;18:37. doi: 10.1186/s13036-024-00432-5 (PMC11157810; doi:10.1186/s13036-024-00432-5)
Supplement: Supplementary file 1 — Supplementary Material 1 [file 13036_2024_432_MOESM1_ESM.docx]

Supplementary Material

**Hypoxia and re-oxygenation effects on human cardiomyocytes cultured on polycaprolactone and polyurethane nanofibrous mats**

**Zuzanna Iwoń^a^, Ewelina Krogulec^b^, Aleksandra Kierlańczyk^a^, Michał Wojasiński^c^, Elżbieta Jastrzębska^a,d^*****

*^a^ Chair of Medical Biotechnology, Faculty of Chemistry, Warsaw University of Technology, Warsaw, Poland*

*^b^ Laboratory of Cell Signaling and Metabolic Disorders, Nencki Institute of Experimental Biology PAS, Warsaw, Poland*

*^c^ Department of Biotechnology and Bioprocess Engineering, Faculty of Chemical and Process Engineering, Warsaw University of Technology, Warsaw, Poland*

*^d^ Centre for Advanced Materials and Technologies, CEZAMAT Warsaw University of Technology, Warsaw, Poland*

** Corresponding author elzbieta.jastrzebska@pw.edu.pl Noakowskiego 3, 00-664 Warsaw, Poland*

**A real-time reverse transcription-quantitative polymerase chain reaction**

Specific human cardiac genes whose expression changes in response to hypoxia or hypoxia with re-oxygenation were selected for study (Table S1). Additionally, specific human cardiac genes whose expression changes during cellular aging.

Table S1: The sequence of primer used.

| Gene | Forward Primer | Reverse Primer |
| --- | --- | --- |
| Human, GAPDH | GTGGACCTGACCTGCCGTCT | GGAGGAGTGGGTGTCGCTGT |
| Human, HIF-1α | TATGAGCCAGAAGAACTTTTAGGC | CACCTCTTTTGGCAAGCATCCTG |
| Human, MAPK4 | CCAATGGCAACTCCGAGTCTGT | GGGTCACTGAAGGAATGGGATC |
| Human, TNNT2 | AAGAGGCAGACTGAGCGGGAAA | AGATGCTCTGCCACAGCTCCTT |
| Human, SCN5A | AGGTCGGAAACCTGGTAAGG | TCCTTACCCATGAAGGCTGTG |
| Human, SERCA2 | GGACTTTGAAGGCGTGGATTGTG | CTCAGCAAGGACTGGTTTTCGG |
| Human, GATA-4 | GCGGTGCTTCCAGCAACTCCA | GACATCGCACTGACTGAGAACG |
| Human, ACTA1 | AGGTCATCACCATCGGCAACGA | GCTGTTGTAGGTGGTCTCGTGA |
| Human, ACTN1 | CAGGACCGTGTGGAGCAGATTG | CAGATTGTCCCACTGGTCACAG |
| Human, MYL2 | CGGAGAGGTTTTCCAAGGAGGA | CTCTTCTCCGTGGGTGATGATG |

**Genes expression for HCM during passage**

*GATA4* and *ACTA1* genes are early cardiomyocyte differentiation markers, *ACTN1* is late HCM differentiation markers, *TNNT2* and *MYL2* are terminal markers of cardiac cell differentiation. These genes were selected to determine mature HCM in depends on the number of passages. An increase in the expression of terminal genes can indicate the aging of HCM cell cultures. Based on the results in Figure S1, it was evaluated that the cells could be used for experiments up to 6-8 passages.


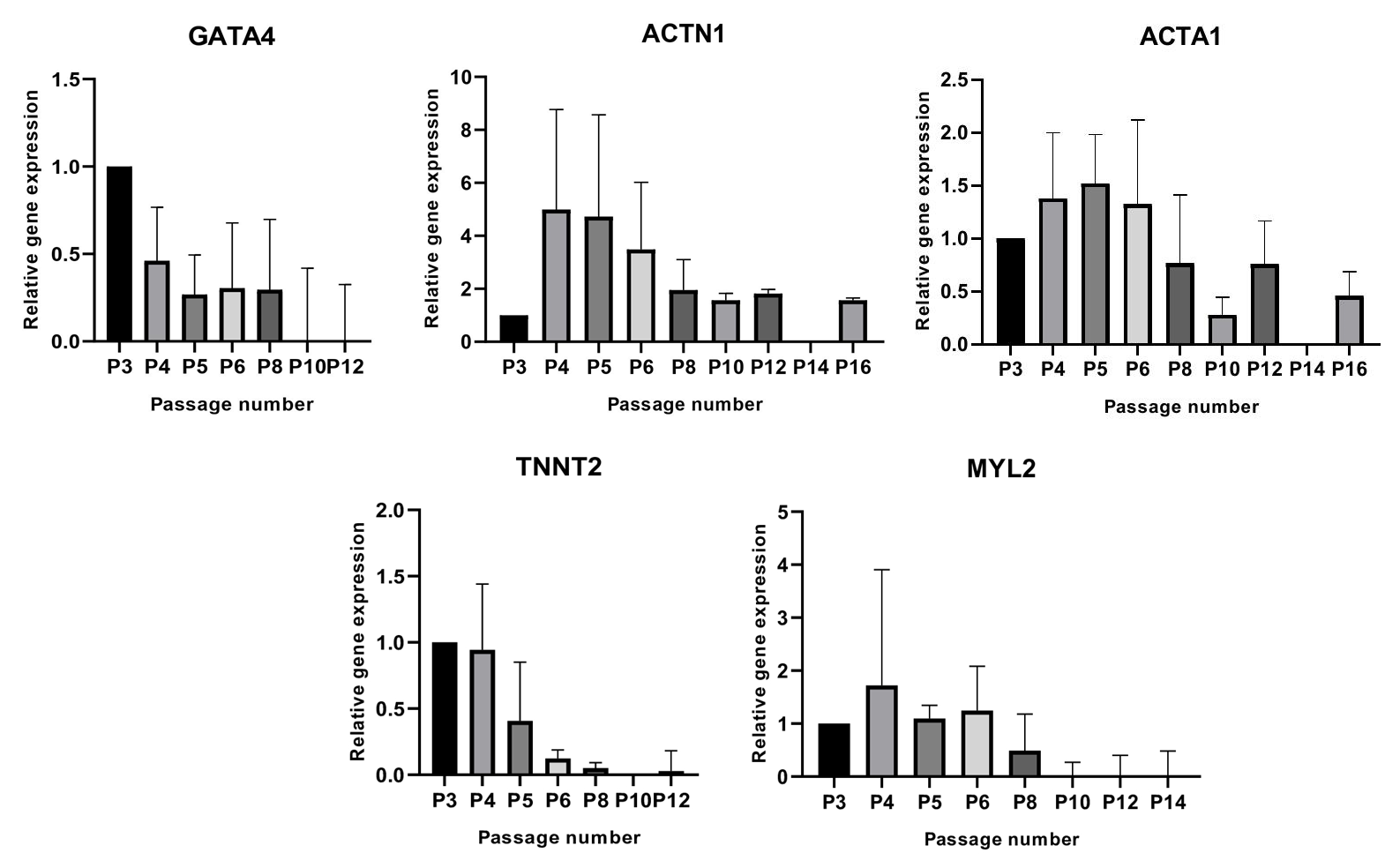


Figure S1: Gene expression analysis of HCM cells after multiple passages. GATA4 encoding GATA binding protein 4, ACTN1 encoding α-actinin 1, ACTA1 encoding α-actin 1, TNNT2 encoding cardiac troponin T, MYL2 encoding myosin light chain 2. n=3.


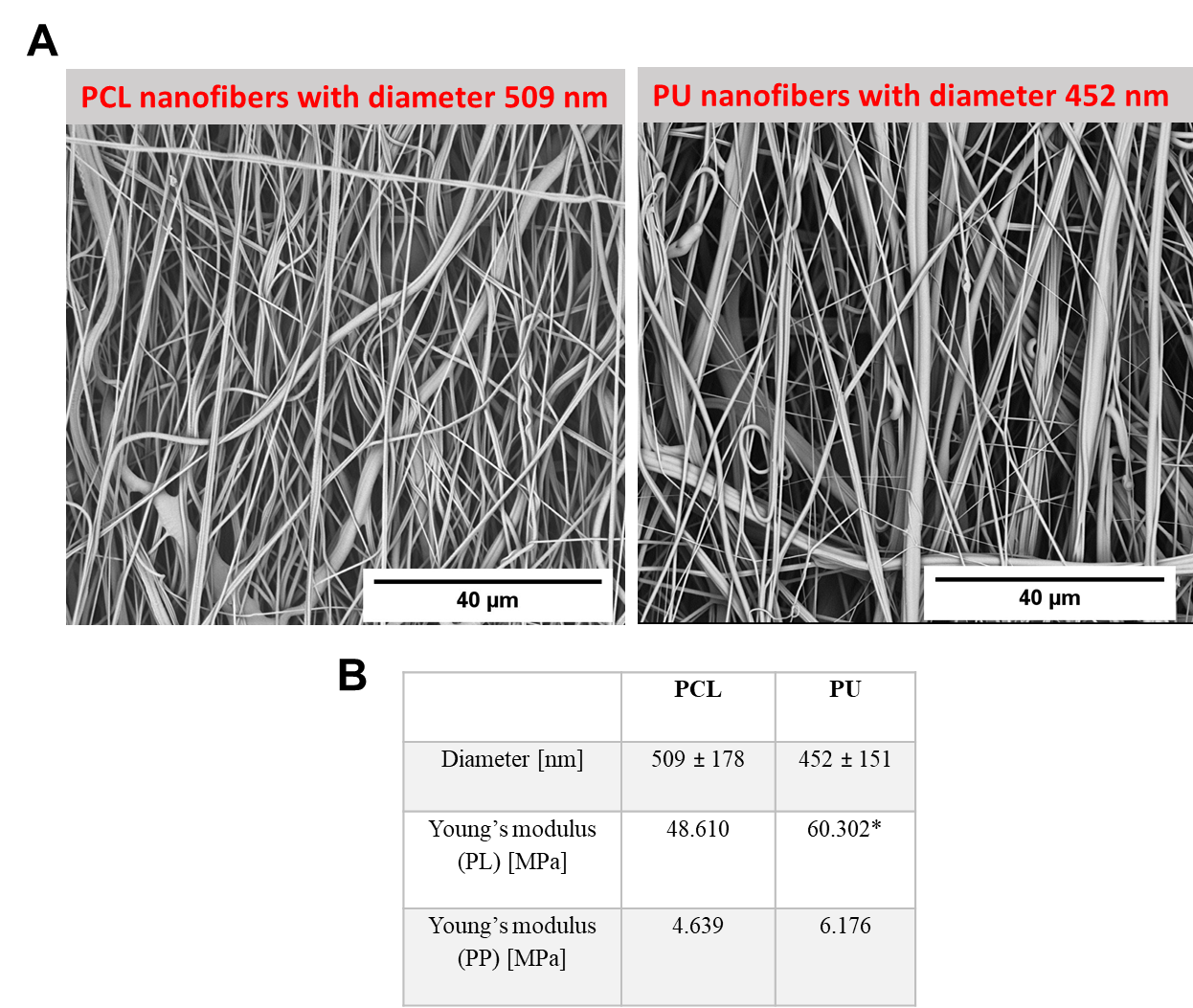


Figure S2: (A) SEM images of aligned PCL nanofibers with an average diameter of 509 ± 178 nm, PU nanofibers with an average diameter of 452 ± 151 nm, (B) Physical properties of polycaprolactone (PCL) and polyurethane (PU) nanofibers. Diameter of nanofibers, and the tensile strength was measured parallel (PL) and perpendicular (PP) to the fiber orientation. Scale bar 40 μm. *- p<0.05- statistically significant differences were determined. n≥3.


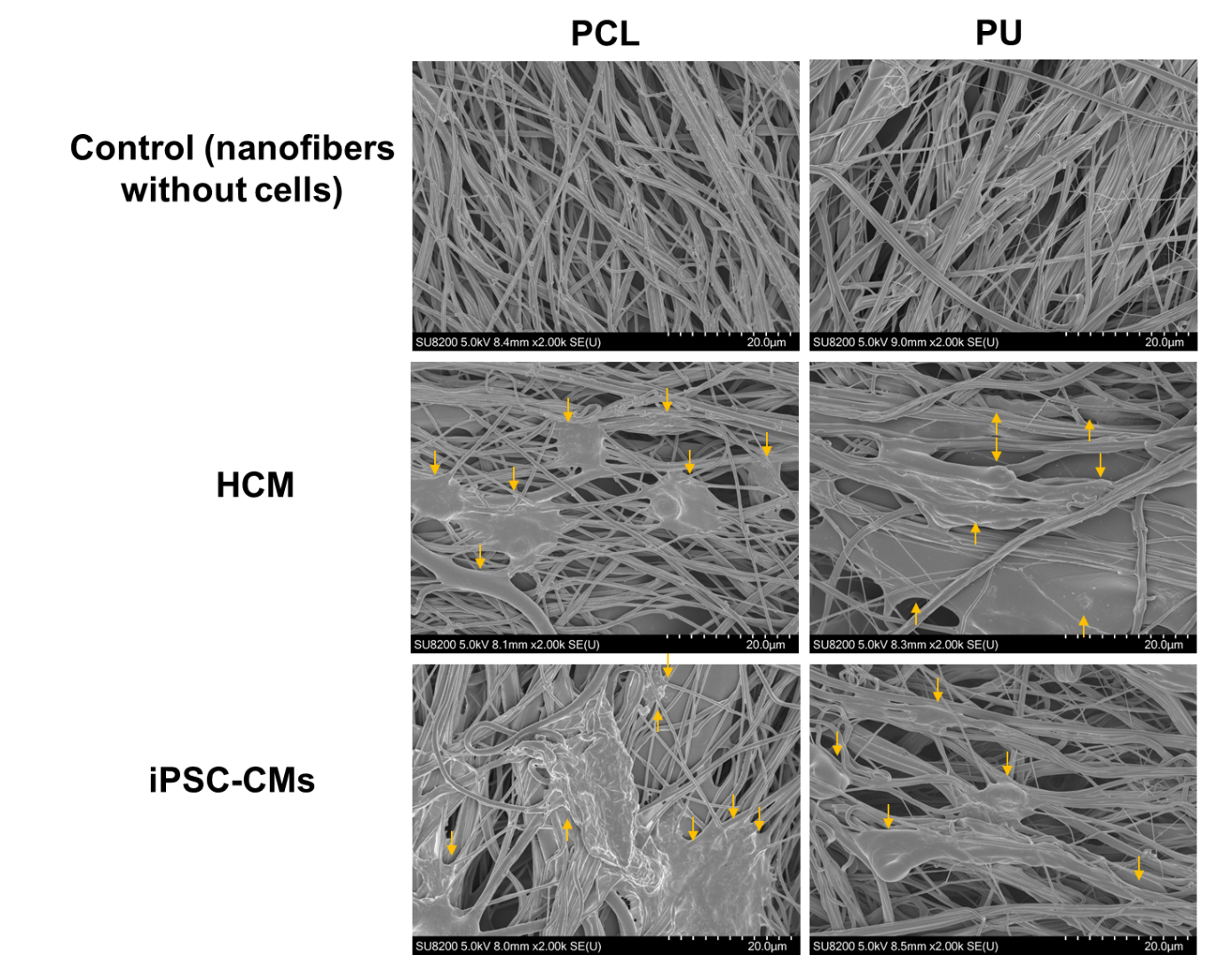


Figure S3: SEM images showing nanofibers without cells (control), HCM, and iPSC-CMs growing on nanofibrous mats after 10 days, scale bars 20 µm. Orange arrows indicate the cells. The images also show cell elongation along the nanofibers.


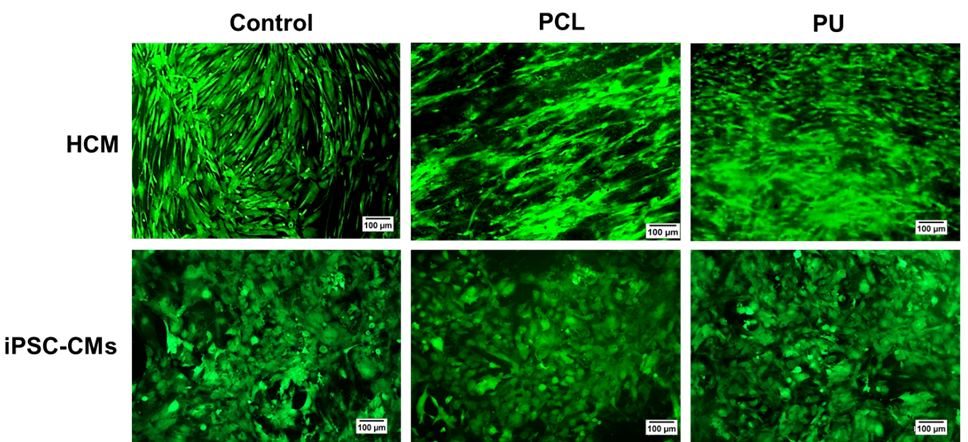


Figure S4: Calcein-AM staining of HCM and iPSC-CMs cells. Staining was performed after 10 days of culture on a polystyrene plate (control) and PCL and PU nanofibrous mats. Scale bar 100 µm.
